# Safety of Hepatitis B Virus Screening in Blood Donors from the Hospital Foundation of Hematology and Hemotherapy of the State of Amazonas (HEMOAM) in the Brazilian Amazon

**DOI:** 10.3390/v16101632

**Published:** 2024-10-19

**Authors:** Rosa Cristina Caldas Belota, Jean de Melo Silva, Eduardo Luiz do Nascimento, Cláudia Maria de Moura Abrahim, Márcia Costa Castilho, José Pereira Moura Neto, Sérgio Roberto Lopes Albuquerque

**Affiliations:** 1 Programa de Pós-Graduação em Hematologia e Hemoterapia do Amazonas (PPGH/UEA/HEMOAM), Manaus 69050-001, Amazonas, Brazil; rcscenf@yahoo.com.br (R.C.C.B.); eduardo.nascimento@hemoam.am.gov.br (E.L.d.N.); claudia.abrahim@gmail.com (C.M.d.M.A.); jpmn@ufam.edu.br (J.P.M.N.); 2 Programa de Pós-Graduação em Imunologia Básica e Aplicada (PPGIBA/UFAM), Manaus 69080-900, Amazonas, Brazil; biomedicojean@mail.com; 3 Fundação de Medicina Tropical Dr. Heitor Vieira Dourado (FMT-HVD), Manaus 69036-110, Amazonas, Brazil; mycastilho13@gmail.com; 4 Programa de Pós-Graduação em Ciências Farmacêuticas (PPGCF/UFAM), Manaus 69080-900, Amazonas, Brazil

**Keywords:** blood transfusion, safety transfusion, epidemiology, hepatitis B

## Abstract

Background: Hepatitis B is an infectious disease of worldwide importance and of great interest to transfusion medicine. The Amazon region has areas of high endemicity, outlining a worrying scenario for transfusion and epidemiological safety. Objective: To analyze the profiles of serological and molecular markers for HBV of blood donors from HEMOAM. Methods: Blood donors with different patterns of reactivity in serological and molecular screening for HBV were tested for viral load by the qPCR method at the reference center for liver diseases in the state of Amazonas. Results: A total of 230,591 donors were tested, with 3104 (1.34%) found reactive for HBV and 2790 (89.9%) found reactive for isolated anti-HBc. Viral load was not detected in 100% of donors reactive only to HBsAg, while 100% of donors with positive anti-HBc and positive HBsAg or HBV NAT demonstrated a detectable viral load. We also detected one case of occult hepatitis B (0.03%) only with reactive HBV NAT and five donors (0.2%) with positive anti-HBc and HBV NAT. Conclusions: With this result, the great importance of the anti-HBc test for the unsuitability of blood donors was verified, as well as the fundamental introduction of the HBV NAT test in screening for hepatitis B in Brazilian blood banks, as this was the only way to detect the viral infection burden in asymptomatic donors who previously would not be treated, which contributed to the maintenance of the endemicity of hepatitis B in the Brazilian Amazon.

## 1. Introduction

According to the World Health Organization (WHO), 257 million people are chronic carriers of the hepatitis B virus (HBV), equivalent to about 3.5% of the global population [1,2]. Despite studies showing a progressive reduction in endemicity rates, the epidemiology of hepatitis B is not homogeneous in the Brazilian territory [3,4].

From the performance of the molecular “nucleic acid test” for the detection of the hepatitis B virus (HBV NAT) in the screening of blood donors, a variable prevalence was found for carriers of viral DNA for HBV in asymptomatic donors negative for hepatitis B surface antigen (HBsAg), which characterized a new clinical form of infection, determined as an occult infection by the hepatitis B virus—HBV (OBI) [5,6].

As for the molecular and serological markers for detecting the hepatitis B virus, according to the evolution of the disease, the nucleic acid test (NAT) is the first reactive test detectable up to 12 days after infection [7]. The first serological marker, HBsAg, appears in acute infection, about 4 weeks after exposure to the virus, declining to undetectable levels within 24 weeks. The antibody against the total HBV nucleus (anti-HBc) determines the presence of antibodies of both the IgM and IgG classes. Anti-HBc IgM, a marker of recent infection, is found in serum up to 32 weeks after infection. However, this marker may be present in the chronic phase when the infection worsens [8].

Anti-HBc IgG It is the marker of past infection that characterizes previous contact with the virus, remaining for life in individuals who have been infected with the hepatitis B virus. HBeAg It characterizes the phase of viral replication and, when reactive, indicates high infectivity. Anti-HBe appears after the disappearance of HBeAg and indicates the end of the viral replication phase [9]. Anti-HBs is a hepatitis B virus surface antigen-specific antibody. It is the only antibody that confers immunity against HBV. This marker is generally present between the first and tenth (1st–10th) weeks after the disappearance of HBsAg and indicates active immunity (previous contact with the virus or vaccine response). Passive immunity is also detected (use of anti-hepatitis B immunoglobulin or the transfer of maternal antibodies during pregnancy) [10].

With the introduction of HBV NAT in the Amazonas blood center, it was possible to detect cases of occult hepatitis B infection (OBI) in the molecular and serological screening of blood donors presenting the following patterns: NAT HBV positive; HBsAg negative; anti-HBc positive or negative. OBI is characterized by the presence of HBV DNA in the circulation of individuals who are serologically negative for HBsAg and harbor viral loads usually below 200 IU/mL or <103 copies/mL.

The number of studies on the prevalence of OBI in regions of high endemicity in Brazil is small. Arraes et al. (2003) found a 2.7% OBI prevalence among anti-HBc-reactive blood donors in Recife, a northeastern city [11]; Silva et al. (2005) found an OBI prevalence of 3.3% among blood donors reactive for anti-HBc in Porto Alegre in southern Brazil [12]; while Araujo et al. (2022) in Belém, State of Pará, revealed an occult HBV infection prevalence of 0.04% from serum samples reagent (positive) to total anti-HBc but non-reagent (negative) to HBsAg and anti-HBs [13].

The screening of blood donors using nucleic acid testing (NAT) was introduced in the mid-1990s in Germany, Austria and Japan, due to the thousands of blood products and components infected mainly by the human immunodeficiency virus and hepatitis B and C, derived from the viral epidemic that occurred at the time [14,15]. In Brazil, it became mandatory in the screening of blood donors in 2014, since screening for HBV in blood donors in Brazilian blood centers is regulated by the Ministry of Health, with HBV NAT, HBsAg and hepatitis B core antibody (anti-HBc) tests being mandatory [16].

According to the technical manual on hepatitis from the Brazilian Ministry of Health, published in 2018, the HBV NAT test detects the presence of viral particles in the acute and chronic phases of hepatitis B, starting at 10 copies/mL of plasma or serum with a specificity above 99% [17,18].

The quantitative test is capable of directly detecting viral DNA, from the highly preserved region of the S protein coding gene, using an automated platform through a mini-pool of six sample nucleic acid testing (MP-NAT) or individual donor nucleic acid testing (ID-NAT) for confirmation of viral presence, with a sensitivity greater than 95%, thus conferring superiority over serological methods [19]. Another important molecular test is the real-time polymerase chain reaction (qPCR) quantitative test used to confirm the presence of HBV DNA, considered the gold standard for the detection of a low viral load and the monitoring of individuals with chronic infection [20,21,22,23].

Moresco et al. (2014), demonstrated an occult hepatitis prevalence of 2.7% in blood donors in the interior of the state of Amazonas, with a non-reactive serological profile for HBsAg and a reagent for anti-HBc using quantitative molecular tests [24]. Reactivity to anti-HBc alone has been reported in different populations, ranging from 1% to 32%. Furthermore, this serological marker exhibits low specificity and high false-positive rates. Consequently, approximately 40% of individuals with this profile require additional confirmatory tests (such as molecular tests) to obtain the final result. [25]. In contrast, several studies have shown that anti-HBc is the only serological marker found in occult infection with NAT-negative HBV, being considered an important complement to transfusion safety [23,24,25].

With the Bio Manguinhos NAT test, the immunological window was reduced to 12 days for the human immunodeficiency virus (HIV), hepatitis C virus (HCV), and HBV, increasing transfusion safety. The methodological flow follows the following steps: (a) sample preparation in a mini-pool of six or individual samples; (b) extraction of nucleic acid from the biological sample (plasma); (c) nucleic acid amplification; and (d) detection of nucleic acid by real-time PCR [26].

Table 1 summarizes the interpretation of serological and molecular markers for hepatitis B, including the hepatitis B surface antibody (anti-HBs) used to better understand the infectious scenario that can be found in the screening of blood donors, adapted from a recently performed multicenter study.

## 2. Objective

To analyze the profiles of serological and molecular markers for HBV of blood donors from HEMOAM.

## 3. Materials and Methods

A retrospective and descriptive study was carried out using samples from blood donors with positive results in screening tests for hepatitis B: HBsAg, anti-HBc, and HBV NAT. All donors with positive results for these markers were considered deferred, and their blood bags were discarded for transfusion. However, according to the HEMOAM retro-surveillance protocol, in cases of repetition with the same sample and positive/negative or positive/inconclusive results, such donors were called to repeat the tests with a new sample.

The HEMOAM protocol, in its routine, repeats the HBV NAT with an individual sample of all the donors with isolated anti-HBc results. With the results, those reactive patients for HBSAg and HBV NAT were referred to the Dr. Heitor Vieira Dourado Tropical Medicine Foundation-FMT-HVD for repetition of the tests and treatment, since this institution is the reference center for liver diseases in the State of Amazonas.

### 3.1. Ethics Approval and Consent to Participate

This project was approved by the Research Ethics Committees of the Amazonas Hematology and Hemotherapy Hospital Foundation, under CAAE: 17625119.0.0000.0009, and the Tropical Medicine Foundation Dr. Heitor Vieira Dourado, co-participant center of the project, under CAAE: 17625119.0.3001.0005, according to the resolution 466/2012 of the National Health Council, which advocates the guidelines and Regulatory Norms for Research Involving Human Beings.

The term of free and informed consent was obtained from all participants with positive results in the hepatitis B screening who had their samples submitted for viral load testing.

### 3.2. Population, Sample and Data Collection

Whole blood samples were collected in tubes with EDTA anticoagulant from blood donors from December 2014 to December 2018.

### 3.3. Methods for Detection of Hepatitis B

#### 3.3.1. Tests Performed at HEMOAM

The technical procedures were carried out in accordance with the specifications of the reagent and equipment manufacturers, using the commercial kits routinely adopted by HEMOAM.

To perform the HBsAg and anti-HBc tests, the chemiluminescence immunoassay (CMIA) method was used using ARCHITECT HBsAg Qualitative II^®^ kits with a sensitivity of 99.52% and ARCHITECT Anti-HBc II^®^ kits with a sensitivity of 99.10% [28]. To carry out the molecular screening, the qualitative HBV NAT Kit was used, manufactured by the company Bio Manguinhos^®^, FIOCRUZ, belonging to the Ministry of Health of Brazil, which was carried out by the qPCR method using the method of extracting genetic material through magnetic spheres, using a mini-pool of 6 samples (MP NAT) and individual samples (ID NAT), with a limit of detection of 300 IU/mL in MP NAT. It should be noted that the reactive results in the screening for HBV were confirmed by repeating the chemiluminescence tests for HBsAg and anti-HBc with the same methodology in duplicate, while the molecular test for HBV DNA was confirmed with the individual testing of each sample that made up a mini-pool of six reactive samples.

#### 3.3.2. Viral Load Detection Performed at FMT-HDV

The viral load detection protocol performed at FMT-HVD employs the quantitative qPCR methodology using the ABBOTT Real Time HBV^®^ KIT, in accordance with the manufacturer’s recommendations, with a minimum detection limit of 3.41 IU/mL.

### 3.4. Data Analysis

The data obtained were entered into a Microsoft Excel (Office 2016 Professional Plus) spreadsheet for cleaning and exported to statistical analysis software (SPSS Version 19) for descriptive analysis using frequencies. The prevalence of serological and molecular markers and their respective 95% confidence intervals (95% CI) were determined.

## 4. Results

Two hundred and thirty thousand, five hundred and ninety-one (230,591) serological and molecular screening results were analyzed, with 3104 (1.35%) found positive for any HBV marker. It is important to emphasize that all blood donors were asymptomatic individuals, able to donate blood in the hematological and clinical screening. As for reactivity to HBV, the following percentages were detected per indicator: HBV NAT: 94 (3.02%); HBsAg: 308 (9.92%) and anti-HBc: 2.913 (93.8%). Table 2 shows how these indicators combined to form the different reactivity profiles.

Additionally, six cases of occult hepatitis B infection (OBI) were identified, with one case (0.03%) being solely detected through positive molecular screening (HBV NAT) and the remaining five cases (0.2%) being identified through both HBV NAT and positive anti-HBc, which would have been undetectable prior to the implementation of molecular screening (NAT HBV).

## 5. Discussion

In Germany, Houareau and Offergeld (2019) evaluated 31,562,556 blood donors over a 10-year period, observing 68,741 with isolated anti-HBc positive who underwent HBV NAT [29]. Of these, 84 (0.12%) had a detectable viral load. Comparatively, in our study, in 2795 donors reactive for anti-HBc, 5 (0.2%) were also reactive in HBV NAT (HEMOAM) with viral load detection (FMT-HDV).

In some cases, discrepant results were found between the qPCR tests performed at HEMOAM (MP qualitative NAT HBV) and at FMT-HDV (quantitative Abbott), where a donor NAT HBV+ (HEMOAM) presented a negative viral load (FMT-HDV), probably characterizing a phase conversion of the infection. It was also observed that all 12 donors with a NAT (−)/HBsAg (+)/Anti-HBc (+) HEMOAM profile, who sought out the FMT-HDV reference center, that is, 100%, had a detectable viral load; this can be explained due to the low number of viral copies found, demonstrating that the MP HBV NAT test cannot be performed alone in the screening of blood donors for hepatitis B. However, these discrepancies may also be due to the characteristics of the tests, such as difference sensitivity and the minimum detection limit for each test, also considering the presence of variant viruses and the target sequences for amplification of each kit. It is important to highlight that 100% of donors with positive results, confirmed for hepatitis B in all results profiles, were compulsorily notified to the health authority [29,30,31,32].

According to Candotti et al. (2018), a range between 0.09% and 0.29% of donations with an initially positive result may become non-reactive in discriminatory tests. The reasons for this discrepancy remain unclear but reflect the Poisson distribution statistics of HBV DNA levels around the assay readout [25].

Another important point that can be observed in the results of this study is the importance of carrying out anti-HBC in the screening of blood donors, as in profile 5 in Table 2; 100% of profile 5 donors (NAT (−), HBSAg (+), anti-HBC (+)) who tested for FMT-HDV had a detectable viral load, while 100% of profile 7 donors (NAT (−), HBSAg (+), anti-HBC (+) who looked for the reference center did not present it.

Dodd et al. (2018) questioned in their study the use of the HBsAg test in the screening of blood donors because in 22,370,271 donations analyzed over a 5-year period in the United States, the authors found 2987 reactive HBsAg, of which only 144 were reactive in NAT HBV with no other marker detected [33]. They also observed that 47 of these 144 reactive samples had a recent vaccination history. After high-sensitivity serological and molecular assays, the authors concluded that the frequency of HBV in this outcome profile would have an infectious risk of 1 in 4.4 million donations, a strong argument for discontinuing the use of the HBsAg assay in donor screening tests [30]. Kiely et al. (2018) [34] corroborated the study by Dodd et al. (2018) [33], which described as the main cause for the false-positive results of HBsAg the vaccination against influenza, rabies and HBV itself, or due to cross-reactions of the epitopes of HBsAg and viral antibodies resulting from the adjacent immune response.

Moresco et al. (2014) found an overall prevalence of occult hepatitis B among blood donors from the interior of the state of Amazonas of 2.7% [30], and in this study, six cases (0.23%) were found [24]. It is important to emphasize that the samples in the study by Moresco et al. came from the southwest of the state of Amazonas, where there is a high prevalence of hepatitis B, with approximately 100% of donors from this region reactive for anti-HBc. For this reason, HEMOAM stopped collecting blood bags from individuals from this area. In the present study, the donor samples were from cities with a low rate of hepatitis B, which may have contributed to the low frequency of occult hepatitis B.

The NAT (+)/HBsAg (−)/Anti-HBc (−) occult hepatitis B profile was found in only one donor (0.03%), in contrast to the study by Pondé (2013) that considered this profile a relatively common finding in blood banks when contact with HBV was very recent and other serological markers of the infection had not yet emerged, or even by the occurrence of the X-mutant virus, generally characterized by a deletion of eight nucleotides between positions 1770 and 1777 and a point mutation from a thymine to a cytosine in the X-ORF region [32]. This suppression results in a C-terminal X protein, truncated with 134 amino acids (aa) due to a loss of 23aa and the addition of normal 3aa, which causes a loss of its transcriptional activity. The elimination of eight nucleotides from the core promoter/enhancer II complex sequence may decrease the function of this transcription control element, promoting the suppression of viral replication and the health of HBcAg and HBsAg. Consequently, donors infected with a defective HBV X-mutant are negative for HBsAg and Anti-HBc, despite the presence of HBV replication and detectable DNA [35,36].

In summary, the results found in this study are of great significance, as it was possible to prove the reliability of the combination of tests used in serological and molecular screening, mainly HBV NAT and anti-HBc, performed in Brazilian blood centers, with the verification of the impact of different profiles found in blood donations and the confirmation of results with viral load detection in a reference center for hepatitis in the Brazilian Amazon.

In the results presented, it is possible to observe the importance of performing the anti-HBc test in the serological screening of hepatitis B, since in 100% of the positive results with HBsAg and anti-HBc, the viral load was detected, whereas in 100% of the positive results, with only HBsAg alone, the viral load was not detected, probably due to the 20% gray zone adopted by HEMOAM in these tests as a safety margin.

## 6. Conclusions

There was a high prevalence of positive laboratory indicators for HBV in blood donors from the Brazilian Amazon, being the main reason for the unsuitability of blood donors due to infectious causes. Serological and molecular screening (HBV NAT-MP, HBsAg and anti-HBc) has been shown to be safe and reliable, allowing the detection of hepatitis B at different stages of the disease, including possible occult infections and HBV gene variants.

The introduction of HBV NAT in Brazilian blood banks made it possible to detect occult hepatitis B infection in donors who previously only had a positive anti-HBc result in isolation.

Positive anti-HBc tests were shown to be important markers for viral load detection in samples that were also positive for HBsAg and negative for NAT HBV.

## 7. Study Limitations

A limitation of this study is mainly due to the low adherence observed among donors positive for HBV by NAT-HEMOAM in complying with retro-surveillance standards and performing the viral load test using quantitative PCR technology in FMT-HDV (only 51 donors out of 314 positives), which may have hindered the accurate demonstration of the study results, although all donors with positive results are compulsorily notified in the Brazilian health surveillance system.

## Figures and Tables

**Table 1:** Clinical laboratory profile obtained through the results of HBV DNA, HBsAg, and anti-HBc. Adapted from Lelie et al., 2017 [27].

DNA HBV	HBsAg	Anti-HBc	Profile
(+)	(−)	(−)	Pre-HBsAg immunological window period or occult infection HBsAg seroconversion or infection in vaccines Occult infection without serological markers
(+)	(+)	NT **	Acute or chronic infection
(+)	(−)	(+)	Post-HBsAg infection Occult infection with negative anti-HBs or occult infection with anti-HBc alone
(−)	(+)	(+)	Low viral load in HBsAg positive and HBV DNA negative
(+)	(−)	NT **	Non-classifiable

HBV DNA: DNA hepatitis B virus; HBsAg: hepatitis B surface antigen; anti-HBc: total hepatitis B core antibody; NT **: not tested.

**Table 2:** Distribution of the prevalence of hepatitis B virus (HBV) infection from blood donor screening in Manaus-Amazonas.

Infection Profile	Screening for HBV	n	%
1	NAT (+)/HBsAg (+)/Anti-HBc (+)	85	2.73
2	NAT (+)/HBsAg (+)/Anti-HBc (−)	3	0.1
3	NAT (+)/HBsAg (−)/Anti-HBc (+)	5	0.2
4	NAT (+)/HBsAg (−)/Anti-HBc (−)	1	0.03
5	NAT (−)/HBsAg (+)/Anti-HBc (+)	33	1.1
6	NAT (−)/HBsAg (−)/Anti-HBc (+)	2790	89.9
7	NAT (−)/HBsAg (+)/Anti-HBc (−)	187	6.02

n: number of samples. NAT: nucleic acid testing; (−): negative; (+): positive; HBsAg: hepatitis B surface antigen; anti-HBc: antibodies against the core antigen of the hepatitis B virus.

## Data Availability

The full data used to support the findings of this study, including consent terms, electronic files and lab techniques and materials, may be released upon reasonable request to my institutional email (sergiorlalbuquerque@gmail.com–Dr. Sérgio Roberto Lopes Albuquerque).
